# Self-Supervised Learning of Satellite-Derived Vegetation Indices for Clustering and Visualization of Vegetation Types

**DOI:** 10.3390/jimaging7020030

**Published:** 2021-02-08

**Authors:** Ram C. Sharma, Keitarou Hara

**Affiliations:** Department of Informatics, Tokyo University of Information Sciences, 4-1 Onaridai, Wakaba-ku, Chiba 265-8501, Japan; hara@rsch.tuis.ac.jp

**Keywords:** vegetation types, clustering, visualization, Sentinel-2, self-supervised, Autoencoders, convolutional, vegetation indices

## Abstract

Vegetation indices are commonly used techniques for the retrieval of biophysical and chemical attributes of vegetation. This paper presents the potential of an Autoencoders (AEs) and Convolutional Autoencoders (CAEs)-based self-supervised learning approach for the decorrelation and dimensionality reduction of high-dimensional vegetation indices derived from satellite observations. This research was implemented in Mt. Zao and its base in northeast Japan with a cool temperate climate by collecting the ground truth points belonging to 16 vegetation types (including some non-vegetation classes) in 2018. Monthly median composites of 16 vegetation indices were generated by processing all Sentinel-2 scenes available for the study area from 2017 to 2019. The performance of AEs and CAEs-based compressed images for the clustering and visualization of vegetation types was quantitatively assessed by computing the bootstrap resampling-based confidence interval. The AEs and CAEs-based compressed images with three features showed around 4% and 9% improvements in the confidence intervals respectively over the classical method. CAEs using convolutional neural networks showed better feature extraction and dimensionality reduction capacity than the AEs. The class-wise performance analysis also showed the superiority of the CAEs. This research highlights the potential of AEs and CAEs for attaining a fine clustering and visualization of vegetation types.

## 1. Introduction

Vegetation is an integral component of life, and identification and classification of vegetation types provides valuable information for understanding the distribution and dynamics of vegetation as for environmental changes. Spectral reflectance measured from remote sensing platforms provides crucial information on identification and discrimination of vegetation types.

The reflectance measured from remote sensors vary with specific biophysical and chemical attributes such as plant type, leaf pigments, water content, and morphological characteristics of the plant canopy concerned [1,2]. Vegetation indices, arithmetic combination of reflectance in multiple wavelengths, have been derived for detecting the biophysical and chemical attributes of vegetation [3]. The vegetation indices are commonly utilized for monitoring and evaluation of extent and coverage of vegetation types [4,5]. However, a large number of vegetation indices exist in the literature, and large numbers of input variables complicate modelling and prediction, and impairs accuracy, known as the “curse of dimensionality” [6,7]. To cope with this problem, dimensionality reduction techniques, which transform high-dimensional dataset into lower-dimensional representations have been proposed [8,9].

Machine learning is a commonly used technique for interpreting remote sensing images into vegetation parameters. There are a number of machine learning algorithms available for dimensionality reductions. The Random Forests (RFs)—an ensemble of decision trees built by splitting the attributes of the data and averaging the output value of all trees—is one of the effective machine learning algorithms for learning non-linear data interactions [10,11]. The RF algorithm also provides an effective statistical measure for determining variable importance [12,13,14]. Researchers have utilized the RFs-based retrieval of important variables as a measure of reducing the dimensions of data [15,16,17,18] and classification of land cover and vegetation types [19,20,21].

Some other classical techniques of dimensionality reductions are principal component analysis [22,23,24], t-distributed stochastic neighbor embedding [25,26], and modified stochastic neighbor embedding [27] as some examples. Oliveira et al. [28] assessed the performance of classical techniques and proposed fractal-based algorithm to remove the redundant attributes accurately.

Artificial neural networks (ANNs) has demonstrated effectiveness in a number of climate change and ecological studies, such as change detection [29], plant identification [30,31], modeling the distribution of vegetation in past, present, and future climates [32], estimation of standing crop and fuel moisture content [33], and mixture estimation for vegetation mapping [34] as some examples.

In recent years, the use of Autoencoders (AEs) has attracted increasing attention to create low-dimensional projections of high-dimensional data. AEs are artificial neural networks (ANNs) designed for learning self-supervised latent representations of multi-dimensional data [35,36,37].The AEs provides a latent-space representation with a reduced dimensionality through the process of compressing (encoding) and decompressing (decoding) of the multi-dimensional data [38,39].

The major objective of this paper is to present an Autoencoders (AEs) and Convolutional Autoencoders (CAEs)-based self-supervised learning approach for the decorrelation and dimensionality reduction of high-dimensional vegetation indices derived from satellite observations. The compressed images are utilized for the clustering and visualization of vegetation types, and they were compared over the Random Forests-based important features. The potential of this approach for the classification of vegetation types is also assessed using the Random Forests (RFs) classifier.

## 2. Materials and Methods

### 2.1. Study Area

**Figure 1:**
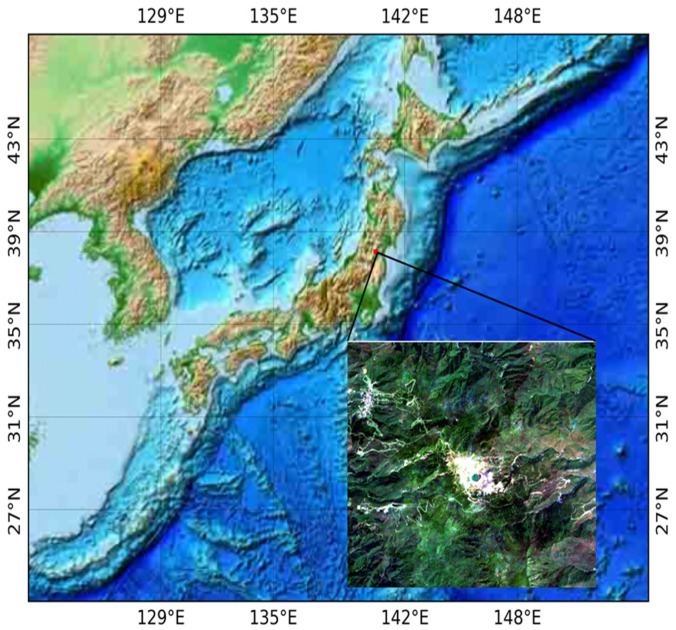
Location of the study area, Mt. Zao, and its surrounding base, shown by a true-color composite image, generated from Sentinel-2 data.

This research was implemented in Mt. Zao, which is located on the border between Yamagata and Miyagi prefectures in Japan. This region is characterized by a cool temperate climate with snowfall during winter. It represents a typical mountainous ecosystem in northeastern Japan. The location of the study area is shown in Figure 1.

### 2.2. Collection of Ground Truth Data

The performance of Autoencoders (AEs) and Convolutional Autoencoders (CAEs) for the clustering and classification of vegetation types was assessed with the support of ground truth data. The ground truth data were collected through a field survey, which was conducted in 2018. The field survey was assisted by time-lapse images available in Google Earth. For each vegetation type, 107–300 sample points (longitudes and latitudes), representing a homogenous area of at least 30 × 30 m, were collected. This research dealt with the following list of vegetation types (Table 1) present in the study region.

### 2.3. Processing of Satellite Data

Sentinel-2 scenes available for the study area from 2017 to 2019 (total 343 scenes) were processed. All images were processed for cloud removal and atmospherically corrected to obtain top of canopy reflectance using the Sen2Cor software (v2.8). For each Sentinel-2 scene, 16 vegetation indices (as shown in Table 2) were calculated, and the resulting vegetation index images were composited by computing monthly median values. In this manner, we obtained a monthly stack of vegetation index images, consisting of 192 (16 vegetation indices × 12 months) layers.

### 2.4. Dimensionality Reduction

**Figure 2:**
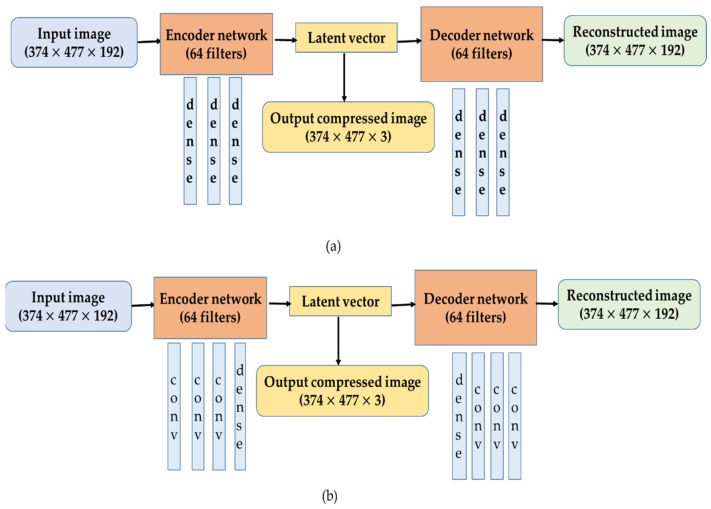
Model architectures: (**a**) Autoencoders (AEs) and (**b**) Convolutional Autoencoders (CAEs) employed in the research.

We employed densely connected Autoencoders (AEs) and Convolutional Autoencoders (CAEs) for the decorrelation of high-dimensional vegetation indices. The model architectures utilized in this research are illustrated in Figure 2. The 192-dimensional stack of vegetation indices was fed into AEs and CAEs models. The AEs were composed of three dense layers; whereas the CAEs were composed of three convolutional layers, and a fully connected (dense) layer was used to collect the outputs from the final convolutional layer. Finally, multiple (3, 5, and 10) low-dimensional latent vectors were obtained from the final dense layer. For the self-supervised learning, we split the dataset into training (95%) and testing (5%) to tune the parameters and hyper-parameters of the models such as the learning rate, number of epochs, and batch size through a repeated trial and error process.

### 2.5. Quantitative Evaluation

The performance of AEs and CAEs-based compressed images for the clustering and visualization was compared to the classical RFs-based retrieval of the important features. The RFs algorithm has been employed as a classical approach for deriving variable importance [55]. The pixel values, corresponding to the ground truth (geolocation points) data, for each vegetation type were extracted from the compressed images (AEs, CAEs, and RFs) and utilized for the visualization and classification of vegetation types. We used 3D scatter plots to visualize the clusters of vegetation types and employed the RFs classifier for the classification of vegetation types.

**Figure 3:**
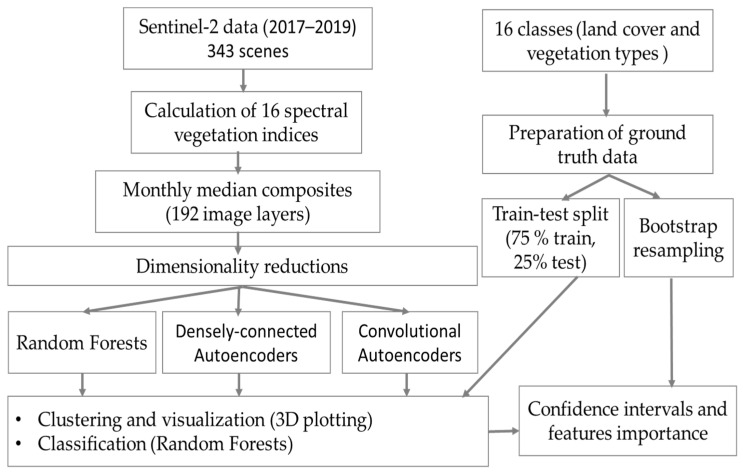
Illustration of the research procedure.

Furthermore, performance of the compressed images (AEs, CAEs, and RFs) in different dimensions (3, 5, and 10) in terms of classification of vegetation types was also assessed quantitatively. For the supervised classification, Random Forests (RFs) classifier was employed on a 75% training set and validated on a 25% test set. For the quantitative evaluation, we computed the confidence interval by implementing bootstrap resampling of the dataset at 1000 times. The bootstrap resampling technique involves drawing of sample data repeatedly with replacement from a data source and reduces a biased estimation of the accuracy. The research procedure has been illustrated in Figure 3.

## 3. Results

### 3.1. Clustering and Visualization

**Figure 4:**
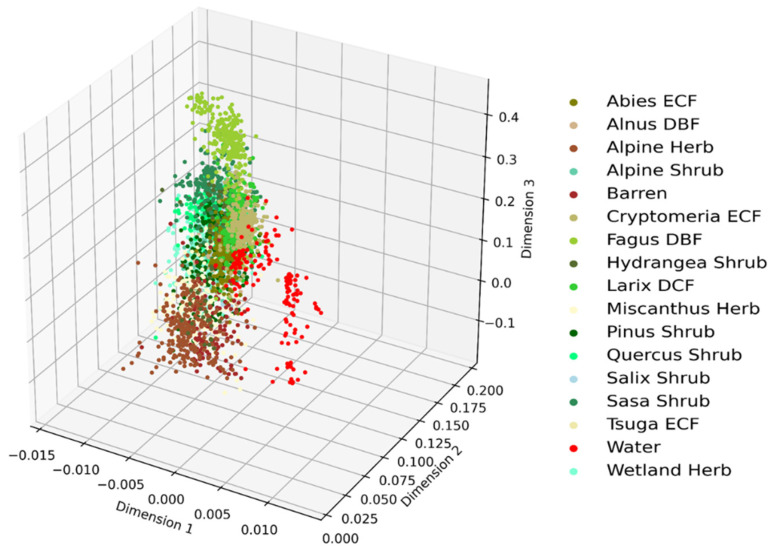
Clusters of vegetation types obtained from Random Forests (RFs)-based important features.

The discriminative ability of the lower dimensional features can be visualized by plotting their distribution in a three-dimensional space. A three-dimensional scatter plot of the RFs algorithm-based retrieval of the most important features is shown in Figure 4. As seen in the figure, most of the inter-class clusters are closed to each other. Therefore, it indicates shortcomings of the RFs-based important features on distinguishing most of the vegetation types.

**Figure 5:**
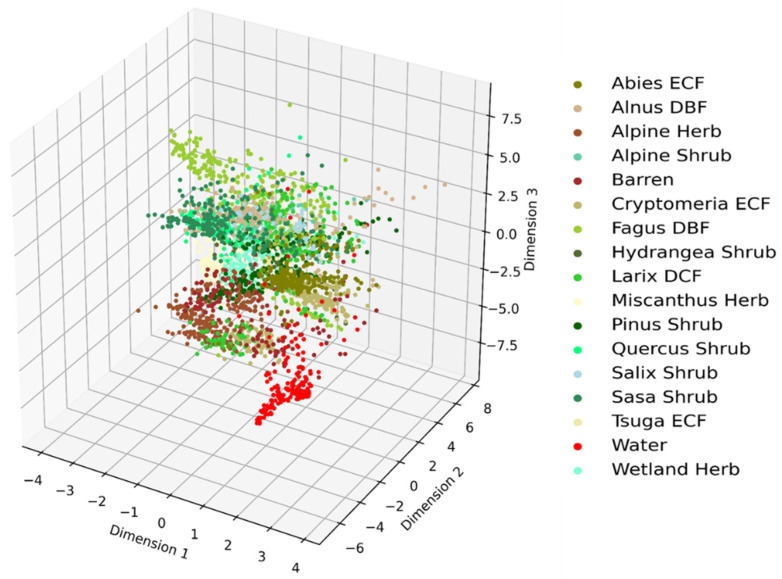
Clusters of vegetation types obtained from Autoencoders (AEs)-based compressed features.

An improvement on the clustering of vegetation types by the AEs-based compressed features over the RFs algorithm can be seen with a wider inter-class variation of the clusters in Figure 5.

**Figure 6:**
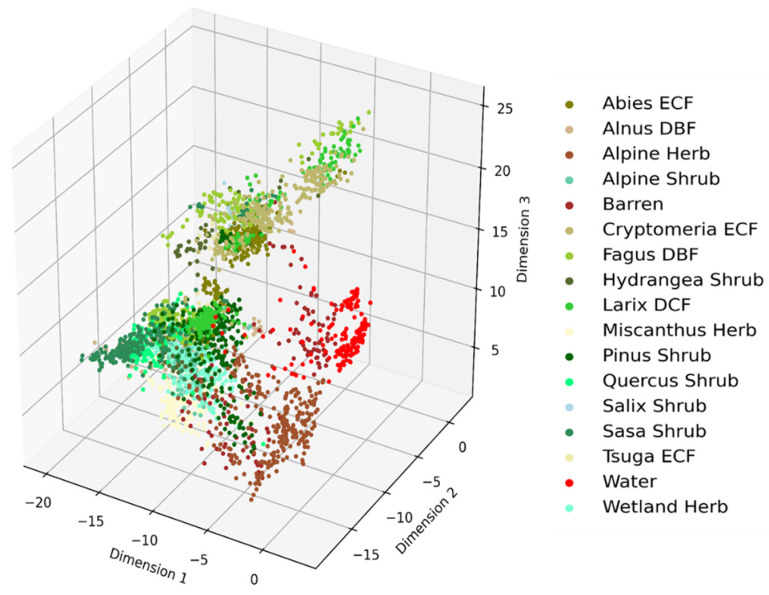
Clusters of vegetation types obtained from the Convolutional Autoencoders (CAEs) model.

Further improvement by the CAEs-based compressed features can be seen in Figure 6. The 3D cluster shows its ability to distinguish vegetation types that were not distinguished by RFs-based important features.

**Figure 7:**
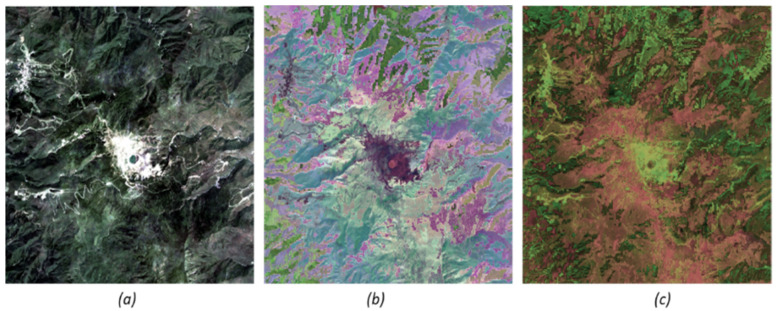
A variation of color shades over different vegetation types (including non-vegetation types): (**a**) Sentinel-2 based true-color composite image, (**b**) Autoencoders (AEs)-based three-dimensional compressed image, (**c**) Convolutional Autoencoders (CAEs)-based three-dimensional compressed image.

The RGB color composites of the AEs and CAEs-based three-dimensional compressed images in Figure 7 demonstrate a variation of color shades over different vegetation types. Generating distinct color shades for different vegetation types under the study is crucial for the improved discrimination and classification with the least number of input features.

### 3.2. Confidence Intervals

**Figure 8:**
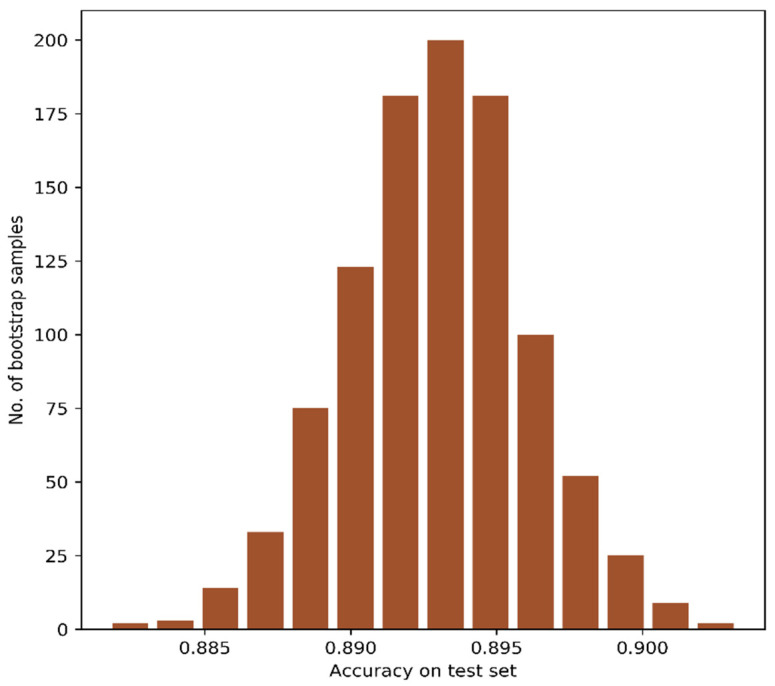
Distribution of test accuracies with bootstrap resampling of CAEs-based three features.

We employed the bootstrap resampling method to report the confidence interval of the CAEs-based classification approach. The bootstrap resampling was done for 1000 times with 75% training and 25% testing data. The accuracy obtained with the test data was collected for each bootstrap resampling, and the frequency of models yielding the test accuracies has been plotted in Figure 8. We also computed the accuracy at a 95% confidence interval. The CAEs-based three features provided test accuracy between 88.7% and 89.9% with a 95.0% confidence interval.

**Figure 9:**
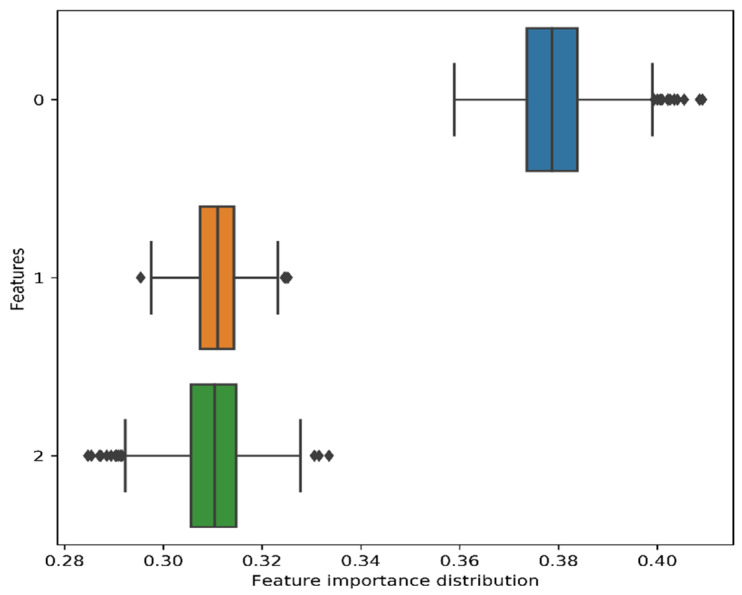
Distribution of feature importance (three features) obtained from the bootstrap resampling.

The distribution of feature importance obtained from bootstrap resampling of the CAEs-based three features has been shown in Figure 9. For each bootstrap resampling, the features distribution showed positive contribution to the model.

**Figure 10:**
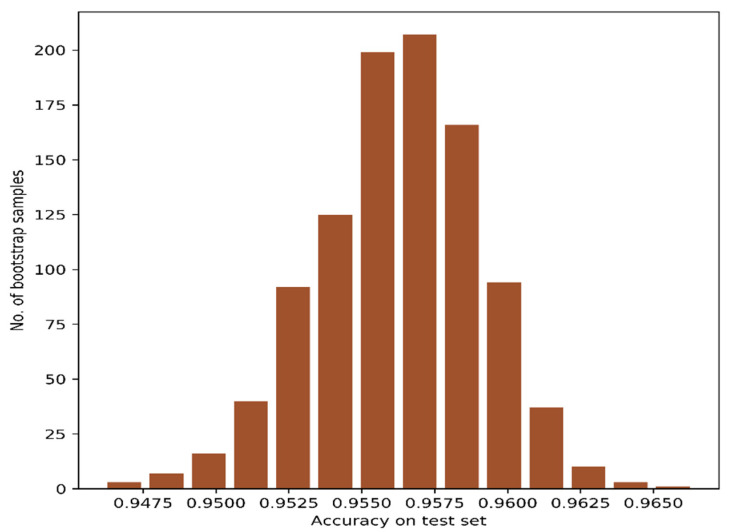
Distribution of test accuracies with bootstrap resampling of CAEs-based ten features.

**Figure 11:**
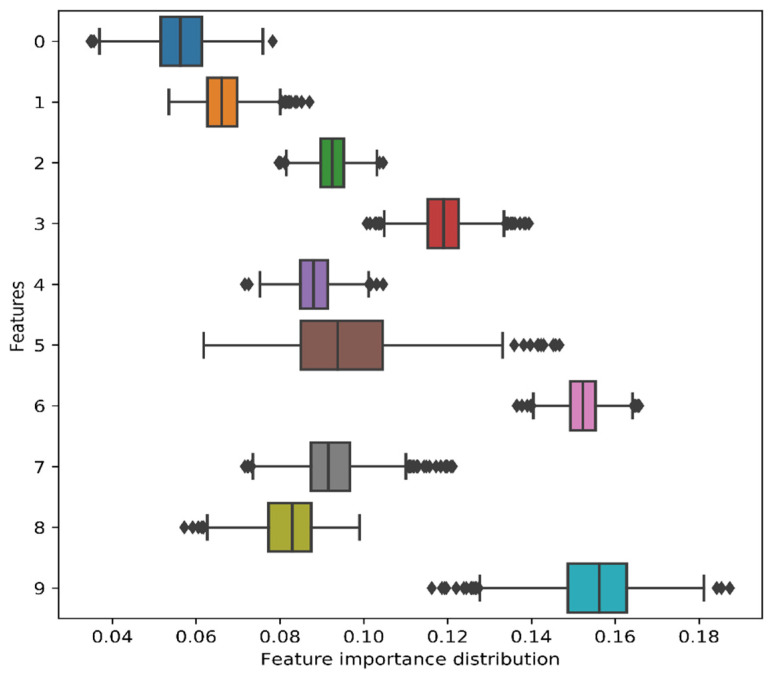
Distribution of feature importance (10 features) obtained from the bootstrap resampling.

Similarly, we calculated the test accuracy using ten features obtained from the CAEs, and the frequency of models yielding the test accuracies has been plotted in Figure 10. The CAEs-based ten features provided test accuracy between 95.0% and 96.2% with a 95.0% confidence interval. In addition, for each bootstrap resampling, the features distribution (10 features) showed positive contribution to the model (Figure 11).

Furthermore, we summarized the significance of the Autoencoders (AEs) and Convolutional Autoencoders (CAEs) over the Random Forests (RFs) by employing bootstrap resampling at 1000 times with 75% training and 25% testing data. Table 3 shows test accuracies computed with a 0.95 confidence interval.

The test accuracies obtained from the bootstrap resampling also showed a higher performance of the Autoencoders (AEs) and Convolutional Autoencoders (CAEs) over the Random Forests (RFs). Interestingly, it should be noted that difference between them (RFs versus AEs or CAEs) started to decrease when number of input features increased. However, the main objective of this research was to compress the high-dimensional dataset into least dimension so as to visualize the inter-class variability of the vegetation types. Therefore, self-supervised learning with the Autoencoders (AEs) and Convolutional Autoencoders (CAEs) has met our objective of showing the inter-class variability of vegetation types at lower dimension. The collection and preparation of ground truth data is very time-consuming and expensive for the vegetation mapping projects. The ability of such self-supervised learning and visualization of the satellite images should contribute to the better interpretation and discrimination of vegetation types (such as collection of ground truth data) as well as subsequent supervised classification for the operational mapping of vegetation types at a broad scale.

## 4. Discussion

We implemented the Autoencoders (AEs) and Convolutional Autoencoders (CAEs)-based self-supervised learning approach for the decorrelation and dimensionality reduction of high-dimensional satellite-based features. Deep learning is a versatile technology specialized for big datasets. Once the high-dimensional features were compressed into lower ones, we employed Random Forests (RFs) classifier for the classification of vegetation types.

A significant processing challenge exists with an ever-increasing collection of huge volumes of remote sensing data with enhanced spatial and spectral resolution. To address this issue, dimensionality reduction techniques have been recommended for reducing the complexity of the data while retaining the relevant information for the analysis [56,57]. Therefore, dimensionality reduction of high-dimensional vegetation indices is a relevant technique, while a large number of vegetation indices exist in the literature.

Spectral vegetation indices have been used by many researchers for the clustering and classification of vegetation types. For example, Villoslada et al. [58] highlighted the need to utilize a wide array of vegetation indices for the improved classification of vegetation types in coastal wetlands. Similarly, Kobayashi et al. [59] utilized spectral indices calculated from a Sentinel-2 multispectral instrument for crop classification. Wang et al. [60] used Fourier transforms on multi-temporal vegetation indices for unsupervised clustering of crop types. These researches motivated us to conduct the clustering and classification of sixteen vegetation types (including non-vegetation classes) solely based on vegetation indices.

Previous studies have also attempted dimensionality reduction of remote sensing data for the classification and mapping of vegetation types. However, most of these researchers have employed classical dimensionality reduction techniques. For example, Alaibakhsh et al. [61] used Principal component analysis (PCA) to delineate riparian vegetation from Landsat multi-temporal imagery. Similarly, Dadon et al. [62] used an improved PCA-based classification scheme to classify Mediterranean forest types in an unsupervised way. The t-Distributed Stochastic Neighbor Embedding (t-SNE) algorithm has been used to strengthen the quality of ground truth data used in the mapping of heterogeneous vegetation [63]. Some researchers have used self-organizing feature maps for the classification of crop types [64,65]. In this context, exploring the potential of deep, self-supervised learning approaches for the clustering and visualization of vegetation types is a timely and important research.

## 5. Conclusions

In this research, we showed Autoencoders (AEs)-based self-supervised learning as a potential approach for the decorrelation and compression of high-dimensional vegetation indices in a cool temperate mountainous ecosystem in Japan. Compared to the classical Random Forests (RFs)-based dimensionality reduction method, the Autoencoders (AEs) and Convolutional Autoencoders (CAEs) showed superior performance on the clustering and classification of vegetation types. While the purpose of dimensionality reduction approaches is to represent the relevant information into the least amount of dimensions, the three-dimensional compression of vegetation indices using the CAEs method showed around a 9% increase in the confidence interval over the RFs. The RFs extracts the most important features out of given features, whereas the AEs and CAEs generate compressed features through self-supervised learning approach. Therefore, this research highlights the application of the CAEs method for the clustering and visualization of vegetation types. In the future, we will assess the efficiency of CAEs in other regions.

## Figures and Tables

**Table 1:** List of vegetation types (including some non-vegetation classes) and size of ground truth data collected.

Vegetation Types	Ground Truth Data Size
(1) Abies Evergreen Conifer Forest (ECF)	300
(2) Alnus Deciduous Broadleaf Forest (DBF)	300
(3) Alpine Herb	300
(4) Alpine Shrub	300
(5) Barren-Built-up area	300
(6) Cryptomeria-Chamaecyparis Evergreen Conifer Forest (ECF)	300
(7) Fagus-Quercus Deciduous Broadleaf Forest (DBF)	300
(8) Hydrangea Shrub	165
(9) Miscanthus Herb	300
(10) Pinus Shrub	300
(11) Quercus Shrub	300
(12) Salix Shrub	108
(13) Sasa Shrub	300
(14) Tsuga Evergreen Conifer Forest (ECF)	107
(15) Water	300
(16) Wetland Herb	300

**Table 2:** List of vegetation indices calculated based on reflectance at Blue (B, Band 2), Green (G, Band 3), Red (R, Band 4), Red edge1 (RE1, Band 5), Red edge3 (RE3, Band 7), and Near infrared (N, Band 8).

Vegetation Indices	Formula	References
(1) Atmospherically Resistant Vegetation Index (ARVI)	$\frac{N-R-\left(R-B\right)}{N+R-\left(R-B\right)}$	Kaufman and Tanre [40]
(2) Enhanced Vegetation Index (EVI)	$2.5\times \frac{N-R}{\left(N+6\times R-7.5\times B\right)+1}$	Huete et al. [41]
(3) Green Atmospherically Resistant Index (GARI)	$\frac{N-\left(G-1.7\times \left(B-R\right)\right)}{N+\left(G-1.7\times \left(B-R\right)\right)}$	Gitelson et al. [42]
(4) Green Chlorophyll Index (GCI)	$\frac{N}{R}-1$	Gitelson et al. [43]
(5) Green Leaf Index (GLI)	$\frac{\left(G-R\right)+\left(G-B\right)}{\left(2*G\right)+R+B}$	Louhaichi et al. [44]
(6) Green Normalized Difference Vegetation Index (GNDVI)	$\frac{N-G}{N+G}$	Gitelson and Merzlyak [45]
(7) Green Red Vegetation Index (GRVI)	$\frac{G-R}{G+R}$	Falkowski et al. [46]
(8) Modified Red Edge Normalized Difference Vegetation Index (MRENDVI)	$\frac{RE3-RE1}{RE3+RE1-2\times B}$	Sims and Gamon [47]
(9) Modified Red Edge Simple Ratio (MRESR)	$\frac{RE3-B}{RE1-B}$	Sims and Gamon [47]
(10) Modified Soil Adjusted Vegetation Index (MSAVI)	$\frac{2N+1-\sqrt{{\left(2N+1\right)}^{2}-8\left(N-R\right)}}{2}$	Qi et al., 1994 [48]
(11) Normalized Difference Vegetation Index (NDVI)	$\frac{N-R}{N+R}$	Rouse et al. [49]
(12) Optimized Soil Adjusted Vegetation Index (OSAVI)	$\frac{\left(NIR-Red\right)}{\left(NIR+Red+0.16\right)}$	Rondeaux et al. [50]
(13) Red Edge Normalized Difference Vegetation Index (RENDVI)	$\frac{RE3-RE1}{RE3+RE1}$	Gitelson and Merzlyak [51]
(14) Soil-Adjusted Vegetation Index (SAVI)	$\frac{1.5\times \left(N-R\right)}{N+R+0.5}$	Huete [52]
(15) Structure Insensitive Pigment Index (SIPI)	$\frac{N-B}{N-R}$	Penuelas et al. [53]
(16) Visible Atmospherically Resistant Index (VARI)	$\frac{G-R}{G+R-B}$	Gitelson, et al. [54]

**Table 3:** Test accuracies obtained from bootstrap resampling with 0.95 confidence interval.

Features	CAEs	AEs	RFs
3	88.7–89.9%	81.2–85.2%	76.7–81.2%
5	92.7–93.8%	87.9–91.4%	84.4–88.6%
10	95.0–96.2%	91.5–94.6%	90.2–93.7%

## Data Availability

Not applicable.
